# Dual Transcriptional Profile of *Aspergillus flavus* during Co-Culture with *Listeria monocytogenes* and Aflatoxin B1 Production: A Pathogen–Pathogen Interaction

**DOI:** 10.3390/pathogens8040198

**Published:** 2019-10-20

**Authors:** Iliada K. Lappa, Angeliki Maria Dionysopoulou, Spiros Paramithiotis, Maria Georgiadou, Eleftherios H. Drosinos

**Affiliations:** 1 Laboratory of Microbiology and Biotechnology of Foods, Department of Food Science and Human Nutrition, Agricultural University of Athens, Iera Odos 75, 11855 Athens, Greece; 2 Laboratory of Food Quality Control and Hygiene, Department of Food Science and Human Nutrition, Agricultural University of Athens, Iera Odos 75, 11855 Athens, Greece; 3 Laboratory of Food Process Engineering, Department of Food Science and Human Nutrition, Agricultural University of Athens, Iera Odos 75, 11855 Athens, Greece

**Keywords:** *Listeria monocytogenes*, *Aspergillus flavus*, interaction, gene expression, Aflatoxins

## Abstract

The objective of this study was to investigate the effect of growth temperature and co-culture of *Aspergillus flavus* with *Listeria monocytogenes* on the production of Aflatoxin B1 (AFB1) and the transcriptional profile of associated regulatory and biosynthetic genes. The transcription of virulence- and homeostasis-associated genes of *L. monocytogenes* was also assessed. For this purpose, mono- and co-cultures of *L. monocytogenes* strain LQC 15257 and *A. flavus* strain 18.4 were inoculated into Malt Extract broth and allowed to grow for seven days at 25 °C and 30 °C. AFB1 quantification was performed by HPLC analysis and gene expression assessment by RT-qPCR. AFB1 production was lower at 30 °C compared to 25 °C during monoculture and also lower during co-cultures at both temperatures. This was accompanied by downregulation of *aflM*, *aflR*, *aflP*, and *aflS* during monoculture and *aflM* and *aflS* during co-culture at 30 °C. On the other hand, transcription of *prfA, plcA*, *plcB*, *inlA*, *inlB*, *inlJ*, *murE*, *accA*, *acpP*, as well as *fapR*, was not affected. *sigB* gene was downregulated after co-culture with the fungus at 25 °C and *hly* was downregulated after monoculture at 30 °C compared to 25 °C. In this work, the molecular interactions between *A. flavus* and *L. monocytogenes* were studied for the first time, offering a novel insight into their co-occurrence. Monitoring of their toxigenic and virulence potential at the molecular level revealed a complex dynamic in natural ecosystems.

## 1. Introduction

Microorganisms are the elements that contribute to food contamination the most. Food microenvironments are complex ecosystems within which bacteria and fungi strive to be established. Their interactions result in the development of a microecosystem that has a major influence on the fate of pathogenic species [1,2,3]. A large number of fungal and bacterial interactions in food commodities lead to critical behavioral shifts of the microbiome ranging from antagonism to mutualism [4]. In particular, pathogens can co-exist, co-operate, or compete in a niche heterogeneity. Bacteria are capable of producing compounds that could either repress pathogenesis by inhibiting filamentation or enhance fungal virulence determinants [5]. Co-existence of two major pathogens, such as *Aspergillus flavus* and *Listeria monocytogenes*, has been reported in food systems such as dairy products, eggs, meats, and brewer’s grain [6,7,8,9,10]. The interactions between those two pathogens are critical in shaping the food microbial communities and exert a significant impact on the colonization, survival and even pathogenesis of these microorganisms. The presence of pathogenic microorganisms in food or various metabolites, such as toxins produced by microorganisms, can threaten human health. 

*A. flavus* is a saprophytic fungus known as a producer of low molecular weight toxic secondary metabolites including aflatoxin B1 (AFB1) [11]. The International Agency for Research on Cancer (IARC) has classified AFB1 as a class I human carcinogen posing this mycotoxin the most carcinogenic biotoxin in nature [12]. AFB1 has been reported to suppress immune function, induce mutations, cause liver diseases, acute aflatoxicosis, and even death [13,14]. *L. monocytogenes*, is a Gram-positive pathogenic bacterium capable of colonizing a wide variety of food commodities and thus exerts a major concern for food safety. *L. monocytogenes* has been documented as the causative agent of several outbreaks of food-borne listeriosis, highlighting its significance as a food contaminant [15,16]. Due to its ubiquitous distribution, the behavior of *L. monocytogenes* is reported to be defined by its surrounding microorganisms [17]. The effect that food-related microorganisms may have on the growth of *L. monocytogenes* has been assessed in co-culture with several bacteria species [18], nevertheless, the limited knowledge on *L. monocytogenes* interactions with fungi makes this kind of study valuable. Generally, virulent pathogens express various response mechanisms to facilitate infection and the responses of one pathogen can be reshaped in the presence of another pathogen. Moreover, pathogens may incorporate different genes that broaden niche adaptation and increase virulence [19]. *L. monocytogenes* virulence-associated gene detection, as well as the impact of several food constituents on their expression, have been intensively studied [20,21,22,23,24,25]. This kind of information is crucial from a food safety point of view since it allows assessment of high-risk foods based on their potential to promote microbial growth. Additionally, safety concern predictions regarding *L. monocytogenes* behavior in specific food environments could become much more accurate.

Aflatoxins biosynthesis is a complex enzymatic reaction [26]. The genes encoding the respective biosynthetic pathway in *A. flavus* are physically gathered in a 75 Kb gene cluster [27]. To date, many genes have been identified as members of the aflatoxin pathway gene cluster. In particular, this work focuses on the effect of growth temperature and co-culture of *A. flavus* and *L. monocytogenes* on the production of aflatoxin B1 and the transcriptional profile of the associated regulatory (*aflR* and *aflS*) and biosynthetic (*aflM*, *aflD*, *aflP*) genes. In addition, the effect of the co-culture and temperature on the transcription of the virulence-associated genes *prfA*, *sigB*, *hly*, *plcA*, *plcB*, *inlA*, *inlB*, *inlC*, *inlJ* the fatty acid biosynthesis-associated genes *murE*, *accA*, *and acpP*, as well as *fapR*, that is involved in peptidoglycan biosynthesis of *L. monocytogenes* was also assessed. 

The exploitation of behavior responses along pathogen–pathogen interactions is an upcoming area of research. The present research is one of a few studies dealing with the major potential issue of one pathogen enhancing virulence potential of another during co-existence in the same environment. It is also the first study monitoring such interactions at the molecular level. 

## 2. Materials and Methods

### 2.1. Microorganisms and Sample Preparation

*L. monocytogenes* serotype 4b, strain LQC 15257 was used throughout this study. The strain was isolated from a strawberry sample and deposited to the culture collection of the Laboratory of Food Quality and Hygiene, Agricultural University of Athens [16]. Before experimental use, the strain was grown twice in brain heart infusion broth (Biolife, Milan, Italy) at 37 °C for 24 h. Then, overnight *L. monocytogenes* culture (9 log CFU/mL) was used for inoculum preparation as follows: The culture was centrifuged (12,000× *g*, 10 min, 4 °C), washed twice with sterile saline, and after the appropriate serial dilutions, used to inoculate malt extract broth (MEB, per liter: malt extract, 20 g; peptone, 1 g; glucose, 20 g) at 7.0 to 7.5 log CFU/mL. 

Spore suspensions of *A. flavus* strain 18.4 were prepared from seven-day old fungal colonies grown on malt extract agar (MEA) at 25 °C. Fungal spores were harvested by scraping the surface of the mycelium with a sterile glass rod using an aqueous solution of 0.01% Tween 80 (Merck, Schuchardt, Germany). The final conidia suspensions were assessed using a hemocytometer (Brand, Wertheim, Germany) and adjusted by appropriate dilutions to approximately 10^6^ spores/mL. 

Co-culture of the *L. monocytogenes* and the *A. flavus* strains took place in MEB medium at 25 °C and 30 °C for seven days. Sampling took place after seven days of incubation. The respective monocultures were used as control. During co-culture, the *A. flavus* strain developed on the surface of the MEB medium while the *L. monocytogenes* strain throughout the medium. During sampling, mycelia were collected using sterile forceps. All mycelia samples were washed with double-distilled H_2_O (ddH_2_O) and dried properly in Whatman No 2 paper. For the stabilization of RNA, samples were flash-frozen in liquid nitrogen and stored at −80 °C until RNA extraction. The remaining MEB medium was centrifuged (12.000× *g*, 1 min, sample temperature), the biomass was mixed with 200 μL RNAlater® solution (Ambion, Whaltham, MA, USA) and stored at −80 °C until RNA extraction. Four biological replicates were analyzed. Finally, the cell-free supernatant of MEB was used for aflatoxin B1 qualitative and quantitative assessment. 

### 2.2. Qualitative and Quantitative Aflatoxin B1 Determination

The method that was followed is described in detail in Georgiadou et al. [28]. Briefly, cell-free supernatant was initially mixed with five volumes of methanol:water (80:20 v/v), filtered through Whatman No.1 (110 mm diameter) and adjusted at pH 7.2 adding PBS buffer (per liter: KCl, 0.2 g; KH_2_PO_4_, 0.2 g; Na_2_HPO_4_, 1.16 g; NaCl, 8 g). The filtrate was further cleaned by passing slowly through an immunoaffinity column AflaClean (3 mL widebore, LCTech GmbH, Obertaufkirchen, Germany) followed by triple elution with methanol according to the instructions of the manufacturer and collected in an amber tube. The eluate was evaporated to dryness under a gentle stream of nitrogen and stored in a refrigerator in the dark until analyzed. Qualitative and quantitative determination of aflatoxin B1 took place by a reversed-phase HPLC system equipped with a JASCO PU 980 pump and injection system (JASCO MD, USA) with a 100 μL injection loop, an ODS Hypersyl column (4.6 × 250 mm, 5 μm particle size, Thermo Scientific), a photochemical reactor (LCTech) for post-column derivatization and a JASCO FP-920 fluorescence detector supported by Clarity Lite software.

An isocratic mixture of water: methanol: acetonitrile (6:2:2) at a flow rate of 1 mL/min was used as a mobile phase. The solvents of the mobile phase were filtered through 0.2 μm membrane filters and degassed using an in-line multi-channel vacuum degassing module (VWR, Model 2004) prior to the HPLC pump. Aflatoxin B1 was detected and quantified by fluorescence detection at an excitation wavelength of 360 nm and an emission wavelength of 435 nm. The AFB1 quantity was determined by the respective calibration curve, which was prepared using several calibration solutions (10–100 μg/kg). Recovery values derived from spiked samples of mycelium-free MEB medium contained 30 and 100 ng/mL of AFB1. Each sample was analyzed in triplicate.

### 2.3. Gene Expression Assay

RNA extraction for *L. monocytogenes* was performed according to Hadjilouka et al. [24], using the PureLink RNA Mini Kit (Ambion). RNA from *A. flavus* was extracted as described by Lappa et al. [29]. In brief, approximately 10 mg of fungal lyophilized mycelium were grounded to powder and used for nucleic acid extraction. Total RNA isolation was performed using PureLink RNA mini kit (Ambion, Carlsbad, CA, USA) according to the manufacturer’s protocol. Trizol reagent (Ambion, Carlsbad, CA, USA) was used in RNA extraction. Genomic DNA contamination was removed through treatment with Turbo DNase (Ambion, Carlsbad, CA, USA) according to kit instructions. RNA quantification and purity were assessed spectrophotometrically using a NanoDrop spectrophotometer (IMPLEN, Germany). The absence of genomic DNA was ensured through RT-qPCR amplification. cDNA synthesis took place using the SuperScript First-Strand Synthesis System for RT-PCR (Invitrogen, Whaltham, MA, USA) according to the instructions of the manufacturer in a 20 mL final reaction volume using random hexamers (Invitrogen, USA). 

For the relative quantification experiments, real-time qPCR was performed using KAPA SYBR qPCR Kit Master Mix (2×) for ABI Prism (Kapa Biosystems, Boston, MA, USA). Primers and PCR conditions are presented in Table S1. Two RT reactions were performed for each sample containing ca. 0.2 μg RNA each. Each cDNA was used for gene expression assessment. Cycling conditions were: 95 °C for 0.5 min, 60 °C for 0.25 min, and 72 °C for 0.25 min (40 cycles). Melting curve analysis of the PCR products took place by heating to 95 °C for 15 s, then at 60 °C for 1 min and raise to 95 °C at 0.3 °C/sec. Template-free samples were also used at every run as a negative control. 

### 2.4. Statistical Analysis

The Ct values of the target genes *prfA*, *sigB*, *hly*, *plcA*, *plcB*, *inlA*, *inlB*, *inlC*, *inlJ*, *murE*, *accA*, *acpP*, *fapR aflM*, *aflD*, *aflR*, *aflP*, *aflS* and reference genes IGS, *rpoB*, *16S*, *18S*, *cal* and *tub-β* obtained during the RT-qPCR experiments were processed according to Hadjilouka et al. [23]. In brief, the preprocessing of the data through the Grubbs test for outliers took place. Then, the NormFinder application for Excel [30] was used for stability assessment of the reference genes. Fold change was calculated according to Pfaffl [31,32] and converted to their log_2_ values [33]. The conditions used as control and sample are indicated in the respective figures. The relative transcription of a gene was considered as up- or down-regulated when the log_2_ value of the fold change (log_2_ FC) was above 1 or below −1 (*p* < 0.05), respectively, assessed through one-sample *t*-test. The differences between the AFB1 production during growth at 25 or 30 °C, as well as in the presence or not of *L. monocytogenes,* were statistically assessed through one-way ANOVA with post-hoc Tukey’s honestly significant difference procedure (*p* < 0.05). All analyses were conducted using SPSS v15.0 (SPSS Inc., Chicago, IL, USA).

## 3. Results 

### 3.1. Aflatoxin B1 Production by A. flavus under Co-Culture with L. monocytogenes

**Figure 1:**
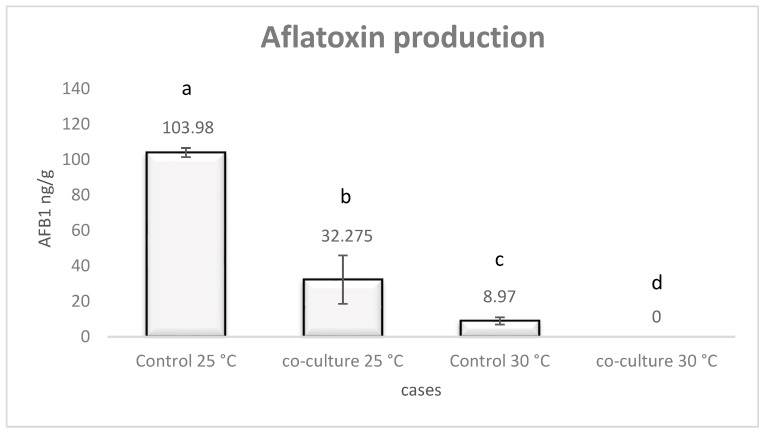
AFB1 production at 25 °C and 30 °C regarding *A. flavus* monocultures and co-cultures with *L. monocytogenes* after seven days of incubation. Error bars represent the standard error of three replicates. Different letters indicate statistically significant differences (*p* < 0.05).

The influence of the *L. monocytogenes* strain LQC 15257 on AFB1 biosynthesis by *A. flavus* strain 18.4 is shown in Figure 1. The fungal isolate was able to produce AFB1 in both temperatures tested. Seven-day incubation was sufficient for AFB1 production by *A. flavus.* In addition, incubation at 25 °C resulted in a higher level of AFB1 production compared to 30 °C. In the presence of the bacterium, the highest amount of toxin was also formed at 25 °C. Co-cultivation with the *L. monocytogenes* strain at both temperatures resulted in a statistically significant (*p* < 0.05) decrease in AFB1 production, compared to the control. The results obtained, showed that *L. monocytogenes* caused greater aflatoxin elimination at 25 °C. However, no aflatoxin was detected after co-cultures at 30 °C. 

### 3.2. Impact of Co-Culture upon Gene Expression

**Figure 2:**
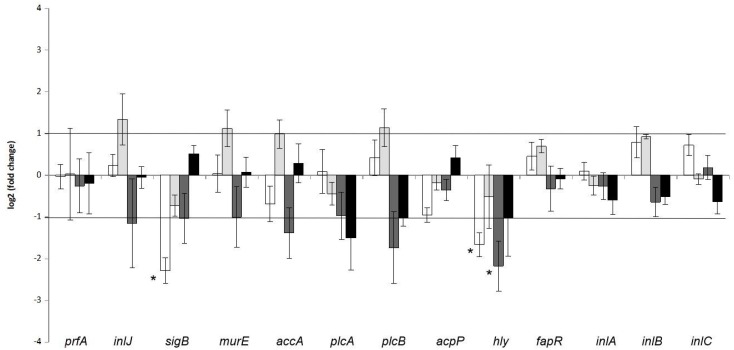
*L. monocytogenes* relative gene transcription after co-culture at 25 °C (control: *L. monocytogenes* monoculture, sample: *L. monocytogenes*-*A. flavus* co-culture, white bars) and 30 °C (control: *L. monocytogenes* monoculture, sample: *L. monocytogenes*-*A. flavus* co-culture, light grey bars), as well as after monoculture (control: *L. monocytogenes* growth at 25 °C, sample: *L. monocytogenes* growth at 30 °C, dark grey bars) and after co-culture (control: *L. monocytogenes*-*A. flavus* co-culture at 25 °C, sample: *L. monocytogenes*-*A. flavus* co-culture at 30 °C, black bars). Error bars represent the standard error of four biological replicates. The asterisk indicates that expression is significantly (*p* < 0.05) above or below the values of 1 and −1, respectively, that were used as threshold.

The effect of co-culture with *A. flavus* strain 18.4 at 25 °C and 30 °C, and temperature, during both monoculture and co-culture on the relative expression of *prfA*, *sigB*, *plcA*, *plcB*, *hly*, *inlA*, *inlB*, *inlC*, *inlJ*, *murE*, *accA*, *acpP* and *fapR* of *L. monocytogenes* strain LQC 15262 is presented in Figure 2. Transcription of eleven genes, namely *prfA*, *plcA*, *plcB*, *inlA*, *inlB*, *inlC*, *inlJ*, *murE*, *accA*, *acpP*, and *fapR* was not affected by the presence of the fungal isolate. It was also observed that temperature change did not present any changes in transcript levels neither in monoculture nor in co-culture with *A. flavus*. On the contrary, *hly* and *sigB* exerted differential regulation. More specifically, both genes were downregulated when *L. monocytogenes* was co-cultured with the fungus but only at 25 °C. In addition, *hly* was downregulated during growth in monocultures at 30 °C compared to 25 °C at monocultures. 

**Figure 3:**
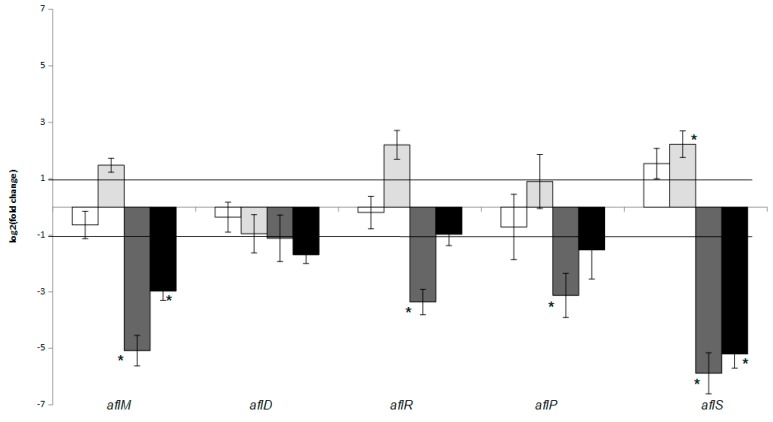
*A. flavus* relative gene transcription after co-culture at 25 °C (control: *A. flavus* monoculture, sample: *L. monocytogenes*-*A. flavus* co-culture, white bars) and 30 °C (control: *A. flavus* monoculture, sample: *L. monocytogenes*-*A. flavus* co-culture, light grey bars), as well as after monoculture (control: *A. flavus* growth at 25 °C, sample: *A. flavus* growth at 30 °C, dark grey bars) and after co-culture (control: *L. monocytogenes*-*A. flavus* co-culture at 25 °C, sample: *L. monocytogenes*-*A. flavus* co-culture at 30 °C, black bars). Error bars represent the standard error of four biological replicates. The asterisk indicates that expression is significantly (*p* < 0.05) above or below from the values of 1 and −1, respectively, that were used as threshold.

In Figure 3, the effect of co-culture with *L. monocytogenes* strain LQC 15262 at 25 °C and 30 °C, and temperature, during both monoculture and co-culture on the relative expression of *aflM*, *aflD*, *aflR*, *aflP*, and *aflS* of *A. flavus* strain 18.4, is shown. Co-culture at 25 °C had no effect on the transcription of the genes under study. On the contrary, co-culture at 30 °C resulted in upregulation of *aflS* gene. The effect of temperature seemed to be more pronounced than the effect of co-culture. More accurately, *aflM*, *aflR*, *aflP*, and *aflS* were downregulated during the growth of the fungus as a monoculture at 30 °C compared to 25 °C. In addition, *aflM* and *aflS* were downregulated during growth of the fungus in co-culture at 30 °C compared to 25 °C. The transcription of *aflD* was not affected by the conditions under study. 

## 4. Discussion

Scarce literature is currently available on bacterial–fungal interactions (BFI) in general and regarding foodborne pathogens, in particular. Such interactions widely exist in distinct microenvironments, including food systems [34]. Their elucidation could provide valuable clues and enable a complete view of regulating mechanisms of a microbiome. Co-culture studies are valuable in understanding co-infection. While individual physiology is well documented, the behavior of pathogens under co-existence is currently unclear. Different holistic omics approaches could reveal various physiological responses during fungal and bacterial co-occurrence, which are probably undetectable by other means [35,36]. In the approach that employed here, transcriptomic analysis of a defined set of genes of both bacterium and fungus was performed, giving the opportunity to monitor changes in gene expression of a microorganism in the presence of another and to correlate the phenotype assessed, i.e., AFB1 production, with gene expression under different conditions. 

In general, aflatoxins biosynthesis is strongly dependent on growth conditions [37,38]. Regarding *A. flavus* and AFB1 production, temperature, among environmental factors, usually has the most incisive effect [39,40]. However, several reports have established different optimal conditions for AFB1 accumulation. These differences found in the literature support the assumption that production is likely due to strain variability or due to different media used. In this study, 25 °C was reported as the optimum temperature for the highest production of AFB1, as Falade et al. also reported in a similar interaction study [41]. AFB1 co-culture findings herein seem to be in accordance with several authors reporting that conditions imposing intermediate stress seem to favor toxin formation, whereas increased stress conditions appeared inhibitory [42,43,44]. Stimulation of mycotoxin production is demonstrated as a typical metabolic response to stressful conditions, which also include the presence of another microorganism [45,46]. Specifically, an increase of AFB1 production in the presence of *L. monocytogenes* has been reported by Asurmendi et al. [47]. However, in the present work, a significant reduction of AFB1 was evident after co-culture with *L. monocytogenes.* In general, the regulatory system of fungal secondary metabolites production can be influenced by a wide range of parameters which are not always associated with growth [48,49]. In fact, in our experiments, we did not observe any statistically significant change in *A. flavus* biomass during co-culture with *L. monocytogenes* (data not shown). This observation is in accordance with Verheecke et al. [48], reporting that growth was not correlated with AFB1 in *A. flavus* during co-culture conditions.

Furthermore, in the present study, the influence of this co-existence in *A. flavus* relative gene expression was also assessed. Specifically, the relative expression of three structural genes, namely *aflD, aflM*, and *aflP* and two regulatory genes, namely *aflR* and *aflS* was studied. In particular, *aflD, aflM* and *aflP* encode enzymes that are necessary for the conversion of norsolorinic acid to averantin, versicolorin A to demethylsterigmatocystin and sterigmatocystin to O-methylsterigmatocystin, respectively [50,51], while *aflR* and *aflS* are the key regulatory genes of AFB1 biosynthetic pathway [52]. Increase of growth temperature negatively affected toxin formation through downregulation of *aflR* and *aflS*, the former during monoculture and the latter during both mono- and co-culture with the *L. monocytogenes* strain. At the same time, the downregulation of *aflM* and *aflP* during monoculture, as well as *aflM* and *aflS* during co-culture, were observed. These results are also in concordance with Verheecke et al. [48] who similarly observed that the expression of *aflM* was repressed by *Streptomyces* presence and was also correlated with AFB1 content. The complex influence that temperature may have on the regulation of *A. flavus* aflatoxin biosynthetic pathway has been previously demonstrated [14,53]. Examining the relationship between the monocultures of *A. flavus* and co-cultures, no difference in gene regulation has been observed at 25 °C. On the contrary, the upregulation of *aflS* was observed at 30 °C. Our observations are resembling similar researches pointing temperature as a modulator of toxin biosynthesis in gene expression level [26,54,55]. Moreover, with regard to regulators, the expression of *aflR* was differently impacted by temperature. Similarly, slight up-regulation of *aflR* and *aflS* in *A. flavus* was observed by Wang et al. [56], when co-cultured at 30 °C presenting however a significant increase of aflatoxin production. Generally, it can be concluded that transcription of regulatory genes *aflR* and *aflS* corresponded to the biosynthesis profile of AFB1 and both biotic and abiotic ecophysiological factors played a key role in gene expression level. It is unclear though if this is a cause of environmental stress, such as temperature effect, or differences in nutritional utilization [55] or any other compound secreted by the bacterial antagonist. Regarding this issue, there are no previous interaction studies to access information about the effect that co-culture of bacterial and mycotoxigenic species may have on the relative gene expression. Barret et al. [35] observed that the plant pathogenic fungus *Gaeumannomyces graminis* triggered gene regulation of *Pseudomonas fluorescence* in the early phases of their interaction. More recently, Benoit et al. [56] reported that *Bacillus subtilis* attachment to *Aspergillus niger* hyphae resulted in mutually altered metabolism. In this study, the co-culture between the microorganisms under study affected only the expression levels of *alfS*. Herein, we concluded that post-transcriptional modifications and other key enzyme interactions may be involved in the suppression of AFB1 formation by *L. monocytogenes*. 

In general, a co-infection by fungi and bacteria may affect their development and concomitantly the outcome of disease [57]. This is also the case of *L. monocytogenes*. The effects of co-existence with other microorganisms on its growth and virulence potential are of great importance, from a food safety point of view, and have been recently reviewed by Zilelidou et al. [17]. In this study, transcription of *hly* was negatively affected by temperature rise during monoculture, as well as during co-culture at 25 °C. Regarding the former, the results obtained in the present study concur with the ones presented by Hadjilouka et al. [34]. In the latter study, it was reported that growth temperature may affect *hly* transcription depending on the growth medium. Indeed, upregulation of *hly* was observed during monoculture in BHI broth at 30 °C compared to 4 °C and 10 °C, while downregulation was observed during monoculture on the surface of rocket salad and melon at the same conditions. These differences were attributed to the complex regulating mechanisms that include three promoter sites, two of which are PrfA-dependent [58], as well as several other factors by different mechanisms [59]. Co-existence with *A. flavus* may be added to these factors, since this is the first time that such a result is presented. Similarly, *sigB* was also downregulated at 25 °C when *L. monocytogenes* was co-cultured with the fungus. *sigB* is encoding a key transcriptional regulator that contributes to the resistance of the pathogen in stressful environments [60,61]. The effect of temperature and food substrate on *sigB* transcription has been studied to some extent [20,21,23]. However, literature is generally lacking data regarding the effect of co-culture with other microorganisms in general and *A. flavus* in particular. Other transcriptional studies have reported various responses of both fungi and bacteria depending on the interacting partners [62,63,64]. These responses may promote different biological outcomes ranging from antagonism to cooperation [65,66].

The effect that bacteria may have on fungal growth, has been studied to some extent in in vitro studies. Usually, interaction studies are conducted in terms of biocontrol either for bacteria or for fungi. However, these interactions may have a different impact on the development of each microorganism [67]. Nevertheless, antagonism is not always the physiological trend of an interaction. This was confirmed by Jung et al. [68]. In the latter study, the authors stated that the protection mechanisms developed against toxins can result in cooperative behaviors between the plant pathogens *B. glumae* and *F. graminearum.*

Until today, making the link between BFIs communication, as in the present work, is a considered significant challenge. However, studies of microbial pathogen interactions are crucial in the field of prediction and food risk assessment including environmental parameters underlying any further potential risks when hazardous microorganisms co-habit in a natural environment. To address the ecological significance of BFI, different experiments have to be conducted in order to determine the nature of interactions that exist between these two pathogens and get further insights into the mechanisms of actions that are involved.

## 5. Conclusions

This work has attempted to investigate the transcription responses of *A. flavus* under co-cultivation with *L. monocytogenes* in a pathogen–pathogen study. The focus was on three structural genes localized in the aflatoxin cluster, as well as two regulatory ones. The transcription of genes associated with aflatoxin biosynthesis by *A. flavus*, as well as *L. monocytogenes* key virulence genes, after co-cultivation of the two microorganisms, was successfully assessed for the first time. Downregulation of *hly* and *sigB* was observed. Both were attributed to the elevated incubation temperature (30 °C compared to 25 °C). In addition, the downregulation of *hly* was also noticed after co-cultivation with the fungus, but only at 25 °C. On the other hand, findings exhibit different gene regulation by *A. flavus* when it co-exists with *L. monocytogenes*, indicating that the fungus was affected by bacterial presence and accordingly, AFB1 biosynthesis reduction was observed. Despite the fact that fungal growth reduction was not observed, AFB1 decreased under co-culture conditions, highlighting alterations of biosynthesis at the molecular level. It is questionable though if the lower level of expression is a consequence or a cause of AFB1 production. However, it should be emphasized that co-culture interactions affected toxin production to a greater extent than the gene expression level. For this reason, additional mechanisms need to be explored since other metabolic pathways can have a significant impact on the complex regulation on the basis of aflatoxin production. Nevertheless, the transcriptional profiles offered the first insight into gene regulation of microorganism interaction related also to their virulence and toxigenic potential.

## Supplementary Materials

The following are available online at https://www.mdpi.com/2076-0817/8/4/198/s1. Table S1. Primer sequences of *L. monosytogenes* and *A. flavus* genes and respective amplicons sizes used for the gene expression assay.
