# Supplementary material for: Dual Transcriptional Profile of Aspergillus flavus during Co-Culture with Listeria monocytogenes and Aflatoxin B1 Production: A Pathogen–Pathogen Interaction
Source: Pathogens. 2019 Oct 20;8(4):198. doi: 10.3390/pathogens8040198 (PMC6963788; doi:10.3390/pathogens8040198)
Supplement: Supplementary file 1 [file pathogens-08-00198-s001.pdf]

Table S1. Primer sequences of *L. monosytogenes* and *A. flavus* genes and respective amplicons sizes used for the gene expression assay

Genes	Primers	Sequence	Concentration (µM)	Amplicon size (bp)	PCR efficiency	Reference
L. monocytogenes						
Reference genes						
IGS	IGS_f	GGCCTATAGCTCAGCTGGTTA	1.2	135	2.03	[1]
	IGS_r	GCTGAGCTAAGGCCCCGTAAA	1.2			
rpob	Rpob_f	CCGCGATGCGAAAACAAT	0.9	69	2.04	[2]
	Rpob_r	CCWACAGAGATACGGTTATCRAATGC	0.9			
16S	16S_f	GATGCATAGCCGACCTGAGA	0.9	114	2.05	[3]
	16S_r	CTCCGTCAGACTTTCGTCCA	0.9			[4]
Virulence- associated						
prfA	prfA_f	CTATTTGCGGTCAACTTTTAATCCT	0.9	100	2.09	[2]
	prfA_r	CCTAACTCCTGCATTGTTAAATTATCC	0.9			
sigB	sigB_f	CCAAGAAAATGGCGATCAAGAC	1.2	166	2.13	[1]
	sigB_r	CGTTGCATCATATCTTCTAATAGCT	1.2			
plcA	plcA_f	CTAGAAGCAGGAATACGGTACA	1.2	115	1.94	[1]
	plcA_r	ATTGAGTAATCGTTTCTAAT	1.2			
plcB	plcB_f	CAGGCTACCACTGTGCATATGAA	0.9	72	2.00	[2]
	plcB_r	CCATGTCTTCYGTTGCTTGATAATTG	0.9			
hly	hly_f	TACATTAGTGGAAAGATGG	1.2	153	1.98	[2]
	hly_r	ACATTCAAGCTATTATTTACA	1.2			
inlA	inlA_f	AATGCTCAGGCAGCTACAMTTACA	0.9	114	2.12	[2]
	inlA_r	CGTGTCTGTTACRTTCGTTTTTCC	0.9			
inlB	inlB_f	AAGCAMGATTTCATGGGAGAGT	0.9	78	2.04	[2]
	inlB_r	TTACCGTTCCATCAACATCATAACTT	0.9			
inlC	InlC_f	ACTGGTCAGAAATGTGTGAATGA	0.9	80	2.06	[4]
	inlC_r	CCATCTGGGTCTTTGACAGT	0.9			
inlJ	inlJ_f	TGCGTAAATGCTCACATCCAAG	0.9	81	2.03	[4]
	inlJ_r	TTGCCCTTCAGCATCCAAGT	0.9			
Fatty acid biosynthesis						
murE	murE_f	GCCACAACCAACAACGACAA	0.9	85	1.97	[5]
	murE_r	TCATACTCCAGACGGCTTGC	0.9			
accA	accA_f	GCGGTCAAAGTGAAGCCATT	0.9	94	1.99	[5]
	accA_r	CCACTTCCACCTTCACCGAT	0.9			
acpP	acpP_f	TGAAGACGAGTTCGGAGTTGA	0.9	91	2.05	[5]
	acpP_r	TGCGTTCGCCTCTATGTACT	0.9			
Peptidoglycan biosynthesis						
fapR	fapR_f	CGCCGTCATCCCAAATGAAA	0.9	88	2.02	[5]
	fapR_r	TGCGATGATGCGTTCTCCTT	0.9			
A. flavus						
Reference genes						
18S	18S_f	GCAAATTACCCAATCCCGACAC	0.2	[illegible]32	1.97	[6]
	18S_r	GAATTACCGCGGCTGCTG	0.2			
cal	cal_f	CTTCCCCGAATTCCTTACC	0.2	[illegible]89	2.02	[7]
	cal_r	TCACGGATCATCTCATCGAC	0.2			
Tub-β	tubβ_f	CGCATGAACGTCTACTTCAACGAG	0.2	[illegible]28	1.94	[8]
	tubβ_r	AGTTGTTACCAGCAGCGGACT	0.2			
Biosynthesis						
aflM	aflM_f	AAGTTAATGGCGGAGACG	0.2	[illegible]72	2.02	[9]
	aflM_r	TCTACCTGCTCATCGGTGA	0.2			
aflD	aflD_f	GCGCAAGTTCCACTTTGAGA	0.2	[illegible]42	1.99	[10]
	aflD_r	CCTTGGTCGCCCATATCAGT	0.2			
aflP	aflP_f	AGCCCCGAAGACCATAAAC	0.2	[illegible]70	1.91	[9]
	aflP_r	CCGAATGTCATGCTCCATC	0.2			
Regulatory						
aflR	aflR_f	CGGCACAGCTTGTTCTGAGT	0.2	[illegible]88	1.92	[10]
	aflR_r	GCATCGTCTCCACCTTCTTG	0.2			
aflS	aflR_f	CTGGCAAAACTTGGGAATGG	0.2	[illegible]63	2.05	[10]
	aflR_r	CACGAGGAAACGGAGTGATG	0.2			

1. Rantsiou, K., Mataragas, M., Alessandria, V., Cocolin, L., 2012a. Expression of virulence genes of *Listeria monocytogenes* in food. *Journal of Food Safety*, **32**, 161-168.
2. Olesen, I., Vogensen, K. F., & Jespersen, L. (2009). Gene transcription and virulence potential of *Listeria monocytogenes* strains after exposure to acidic and NaCl stress. *Foodborne Pathogens and Disease*, **6**, 669-680.
3. van der Veen, S., Abee, T. 2010. Importance of SigB for Listeria monocytogenes static and continuous-flow biofilm formation and disinfectant resistance. *Applied and environmental microbiology*, **76**(23), 7854-7860.
4. Hadjilouka, A., Molfeta, C., Panagiotopoulou, O., Paramithiotis, S., Mataragas, M., Drosinos, E.H. 2016a. Expression of *Listeria monocytogenes* key virulence genes during growth in liquid medium, on rocket and melon at 4, 10 and 30 °C. *Food Microbiology*, **55**, 7-15.
5. Hadjilouka, A., Mavrogiannis, G., Mallouchos, A., Paramithiotis, S., Mataragas, M., Drosinos, E.H. 2017. Effect of lemongrass essential oil on *Listeria monocytogenes* gene expression. *LWT*, **77**, 510-516.
6. Crespo-Sempere, A., Martínez-Culebras, P.V., González-Candelas, L. 2014. The loss of the inducible *Aspergillus carbonarius* MFS transporter MfsA leads to ochratoxin A overproduction. *International Journal of Food Microbiology*, **181**, 1-9.
7. Gerin, D. De Miccolis Angelini, R.M., Pollastro, S., Faretra, F., 2016. RNA-Seq Reveals OTA-Related Gene Transcriptional Changes in *Aspergillus carbonarius*. *PLoS* ONE **11**(1): e0147089.
8. Crespo-Sempere, A., Marin, S., Sanchis, V., Ramos, A.J. 2013. VeA and LaeA transcriptional factors regulate ochratoxin A biosynthesis in *Aspergillus carbonarius*. *International Journal of Food Microbioloy*, **166**, 479–486.
9. Gallo, A., Epifani, F., Bonsegna, S., Pascale, M., Santino, A., Perrone, G. 2010. Analysis of genes early expressed during *Aspergillus flavus* colonisation of hazelnut. *International Journal of Food Microbiology*, **137**(1), 111-115.
10. Gallo, A., Solfrizzo, M., Epifani, F., Panzarini, G., Perrone, G. 2016. Effect of temperature and water activity on gene expression and aflatoxin biosynthesis in *Aspergillus flavus* on almond medium. *International Journal of Food Microbiology*, **217**, 162-169.
